# Mechanical tunability of oriented and random electrospun poly(ε-caprolactone) scaffolds via concentration, molecular weight, and environment

**DOI:** 10.1038/s41598-026-45961-9

**Published:** 2026-03-27

**Authors:** Muhammad A. Munawar, Dirk W. Schubert, Fritjof Nilsson

**Affiliations:** 1 Fibre Science and Communication Network (FSCN) Research Center, Mid Sweden University, Sundsvall, 85170 Sweden; 2 Institute of Polymer Materials, Department of Materials Science and Engineering, Faculty of Engineering, Friedrich-Alexander University Erlangen-Nuremberg, Martensstrasse 7, 91058 Erlangen, Germany; 3 KeyLab Advanced Fiber Technology, Bavarian Polymer Institute, Dr.-Mack-Strasse 77, 90762 Fürth, Germany; 4 School of Engineering Sciences in Chemistry, Biotechnology and Health, Fibre and Polymer Technology, KTH Royal Institute of Technology, Stockholm, 100 44 Sweden

**Keywords:** Electrospun PCL, Fiber alignment, Molecular weight blends, Mechanical properties, Degradation behavior, Engineering, Materials science

## Abstract

Achieving precise mechanical control in electrospun fibrous scaffolds remains a critical challenge for tissue engineering, where scaffold stiffness, strength, and extensibility must be tailored to diverse biological environments. Here, we establish a systematic framework for tuning the mechanical behavior of electrospun poly(ε-caprolactone) (PCL) fibers by integrating molecular-weight blending, polymer concentration control, fiber orientation, and environmental exposure within a single study. High-molecular-weight PCL (H-PCL) and blends with low-molecular-weight PCL (L-PCL) were electrospun to produce fibers with controlled diameters, morphologies, and orientations. Fiber alignment emerged as the dominant structural factor governing mechanical performance: oriented fibers exhibited substantially higher stiffness (~ 90–140 MPa) and tensile strength (up to ~ 100 MPa), while randomly deposited fibers showed markedly greater extensibility (up to ~ 1000%). Polymer concentration and resulting fiber diameter further modulated stiffness, with optimal mechanical performance observed at intermediate concentrations (~ 10–12% w/v). Molecular-weight blending provided an additional route to tailor fiber morphology and modulus, with oriented fibers reaching peak stiffness at ~ 50–60% H-PCL. Environmental exposure studies revealed that acidic treatments (formic and acetic acid solutions) reduce stiffness in a concentration- and temperature-dependent manner, whereas physiological soaking in phosphate-buffered saline (PBS, 37 °C) largely preserves scaffold integrity. Collectively, the electrospun scaffolds developed here span a broad mechanical window (~ 5–140 MPa). When positioned against literature-reported electrospun PCL scaffolds for cardiac, bone, and muscle tissue engineering, this range bridges multiple application-relevant stiffness regimes. These results provide a unified structure–property framework for designing mechanically tunable PCL fibrous scaffolds across diverse biomedical applications.

## Introduction

Poly(ε-caprolactone) (PCL) is a widely used biodegradable aliphatic polyester that has attracted considerable attention in tissue engineering, drug delivery, and regenerative medicine due to its excellent biocompatibility, slow degradation kinetics, and ease of processing^1–4^. Its relatively low glass transition temperature and inherent flexibility make it especially suitable for the fabrication of soft biomaterials, while its stable ester backbone enables long-term structural integrity under physiological conditions^5–7^. Among the many fabrication techniques for PCL, electrospinning has emerged as a versatile method for producing nanofibrous scaffolds that mimic the architecture of native extracellular matrices (ECM). Electrospun PCL fibers combine high surface-to-volume ratio, tunable porosity, and controllable fiber morphology features critical for guiding cell behavior, facilitating nutrient transport, and achieving mechanical performance tailored to specific biomedical applications^8–11^.

A key challenge in designing electrospun PCL scaffolds is understanding the structure–property relationships that govern their mechanical behavior. Mechanical performance depends not only on polymer chemistry but also on processing parameters (e.g., solution concentration, applied voltage, collector configuration), fiber morphology (diameter and alignment), and post-processing conditions^12–15^. Early mechanical studies revealed significant differences between bulk scaffold mechanics and single-fiber properties. For instance, macroscopic mats exhibited Young’s moduli in the megapascal range, while atomic-force microscopy measurements of single fibers indicated moduli in the gigapascal regime. These findings highlight the influence of fiber network architecture and inter-fiber interactions on overall scaffold mechanics. More recent studies have shown that electrospinning parameters, such as applied voltage, can modulate fiber diameter and, consequently, tensile strength, elongation, and elastic modulus, demonstrating that process-induced morphological variations can be used to tune mechanical behavior without altering polymer chemistry^16–18^.

Fiber orientation has been shown to strongly influence mechanical performance. Aligned (or oriented) fibers, collected using rotating mandrels, exhibit anisotropic mechanical properties with superior stiffness and strength along the alignment direction compared to randomly oriented mats. This anisotropy mimics the architecture of native tissues such as tendons, muscle, and vasculature, and enhances load-bearing capacity in engineered constructs^19–22^. For example, aligned PCL/silk fibroin scaffolds demonstrated increased tensile strength and guided stem-cell elongation along the fiber axis compared to random scaffolds^23^. Similarly, hybrid PCL/PLLA electrospun scaffolds showed marked differences in modulus between longitudinal and transverse directions, highlighting the importance of alignment for tissues subject to directional mechanical stimuli^24^.

Beyond fiber morphology, molecular weight and polymer blending critically affect electrospinning behavior and mechanical performance. High-molecular-weight PCL promotes robust chain entanglement necessary for continuous fiber formation but increases solution viscosity, potentially limiting processability^25,23,16^. Low-molecular-weight PCL, in contrast, eases processing but often produces unstable jets due to insufficient chain entanglement. Blending high- and low-molecular-weight PCL has emerged as a strategy to balance processability and mechanical properties. Tailoring the weight fractions can modulate chain entanglement, crystallinity, and chain mobility, influencing fiber formation, stiffness, and ductility. Despite prior studies on PCL blends and co-polymers, systematic exploration of how molecular-weight fractions interact with fiber diameter and orientation to control mechanical behavior remains limited^14,26–28^. For biomedical applications, the degradation behavior of PCL scaffolds is of paramount importance. PCL primarily degrades via hydrolysis of ester bonds, a slow process under physiological conditions due to hydrophobicity and crystallinity. Electrospun scaffolds, however, may encounter more aggressive environments, such as localized acidity or enzymatic activity, particularly in pathological or healing tissues. Physiological degradation is often simulated in vitro by incubating scaffolds in phosphate-buffered saline (PBS) at 37 °C, where modest reductions in stiffness and mass are observed over time^25,29–31^. Molecular-weight distribution and scaffold architecture (e.g., fiber diameter and porosity) influence hydrolytic rates, with lower molecular weight chains and higher surface-area-to-volume ratios accelerating degradation. Acidic environments, in contrast, can rapidly weaken fibers by plasticizing the polymer, disrupting crystallinity, or even dissolving fibers depending on concentration and temperature. Systematic studies exploring acid-mediated degradation in relation to fiber morphology and molecular-weight composition remain scarce^32–35^. Although previous studies have provided valuable insights into electrospinning processes, most investigations primarily focus on the influence of individual solution or processing parameters such as polymer concentration, viscosity, applied voltage, or collector distance on fiber morphology and mechanical behavior. As a result, multidimensional interactions between key variables, including polymer concentration, molecular weight, fiber diameter, and fiber alignment, remain insufficiently explored. In particular, molecular weight blending of high- and low-molecular-weight polymers represents a promising strategy for tailoring the mechanical performance of electrospun scaffolds, yet its combined effects with concentration-dependent fiber formation and orientation control are still poorly understood. Furthermore, degradation behavior under non-physiological acidic environments and mechanical characterization in hydrated or physiological conditions remain limited, despite their relevance for biomedical applications and in *vivo* performance of fibrous scaffolds^36–38^. In tissue engineering, the mechanical properties of scaffolds must be matched to those of the target tissue to ensure appropriate structural support and cellular response. For example, electrospun scaffolds used in cardiac and skeletal muscle applications typically exhibit Young’s modulus values in the range of a few to tens of MPa, whereas bone-related fibrous membranes or reinforced scaffolds often require higher stiffness levels approaching hundreds of MPa. Therefore, the ability to systematically tune the mechanical properties of electrospun fibers is critical for designing scaffolds suitable for different biomedical applications^39,40^.

In this context, the present study systematically investigates how polymer concentration, molecular-weight blending of high- and low-molecular-weight poly(ε-caprolactone) (PCL), and fiber orientation can be used as complementary design strategies to tune the mechanical behavior of electrospun fibrous scaffolds. Mechanical stability and degradation behavior are further evaluated under both physiological and acidic soaking environments to assess the environmental robustness of the produced fibers. By integrating concentration variation, molecular-weight blending, orientation control, and environmental exposure within a single framework, this work establishes a comprehensive structure–property map for electrospun PCL systems. The resulting mechanical window is also contextualized relative to stiffness ranges reported in the literature for cardiac, muscle, and bone tissue engineering scaffolds, highlighting the potential of mechanically tunable PCL fibrous platforms for diverse biomedical applications.

## Materials and methods

### Materials

Two grades of poly(ε-caprolactone) (PCL) with different number-average molecular weights (Mn) were used for electrospinning. A high molar mass PCL (H-PCL; Product No. 440744-500G, Mn ≈ 80,000 g/mol) and a low–molar mass PCL (L-PCL; Product No. 440752-250G, Mn ≈ 10,000 g/mol) were purchased from Sigma-Aldrich (Germany). Both polymers have a density of 1.145 g/mL at 25 °C.

Chloroform/Trichloromethane (TCM, ≥ 99%) and ethanol (EtOH, ≥ 99.8%) were obtained from Carl Roth and used as solvents for polymer dissolution. For soaking and degradation studies, phosphate-buffered saline (PBS; without Ca²⁺ and Mg²⁺) was procured from Sigma-Aldrich. Formic acid (FA, ≥ 98%) and acetic acid (AA, ≥ 99%) were supplied by Carl Roth. All chemicals were used as received without additional purification.

### Electrospinning of PCL solutions

Electrospinning solutions were prepared by dissolving PCL in a mixed solvent system of trichloromethane (TCM) and ethanol (EtOH) at a 4:1 (v/v) ratio. All solutions were magnetically stirred for 2 h at room temperature to ensure complete dissolution and homogeneity. The prepared solutions were transferred into 10 mL glass syringes (inner diameter: 14.65 mm) fitted with an 18-gauge stainless-steel needle. The syringes were mounted on a programmable syringe pump to deliver a continuous and controlled flow of solution during electrospinning. Electrospinning was carried out using a horizontal setup in which the syringe and pump were positioned perpendicular to the rotating collector.

Prior to the experiments, electrospinning parameters were optimized through preliminary trials by varying the applied voltage, feed rate, and tip-to-collector distance to achieve stable Taylor cone formation and continuous fiber deposition without jet instability or bead defects. Although the viscosity of the polymer solutions varied with concentration, a stable electrospinning regime was identified within a defined operating window of the applied electric field. Within this window, the applied voltage was kept constant for all solution concentrations in order to isolate the influence of polymer concentration and molecular-weight blending on fiber morphology and mechanical properties. Minor adjustments in processing conditions during the optimization stage ensured stable jet formation across the investigated concentration range.

All solutions within their respective optimized ranges were subsequently electrospun under fixed processing parameters (Table 1) to obtain uniform fiber mats suitable for mechanical and morphological characterization^41–44^.

**Table 1:** Optimized electrospinning parameters for PCL fiber production.

Syringe radius (mm)	Voltage (kV)	Feed rate (mL/hr)	Tip-to-collector distance (cm)	Collector rotation speed (rpm)
14.65	25	1.0	15	100

### Polymer concentration study

A series of H-PCL solutions were prepared at concentrations ranging from 6% to 24% (w/v) in 2% increments to evaluate the influence of polymer concentration on electrospinnability, jet stability, and fiber morphology. Preliminary trials indicated that fiber formation was strongly governed by solution viscosity and the degree of polymer chain entanglement. Consequently, the effective concentration window for producing continuous, defect-free fibers differed between oriented and random fiber configurations.

**Fig. 1:**
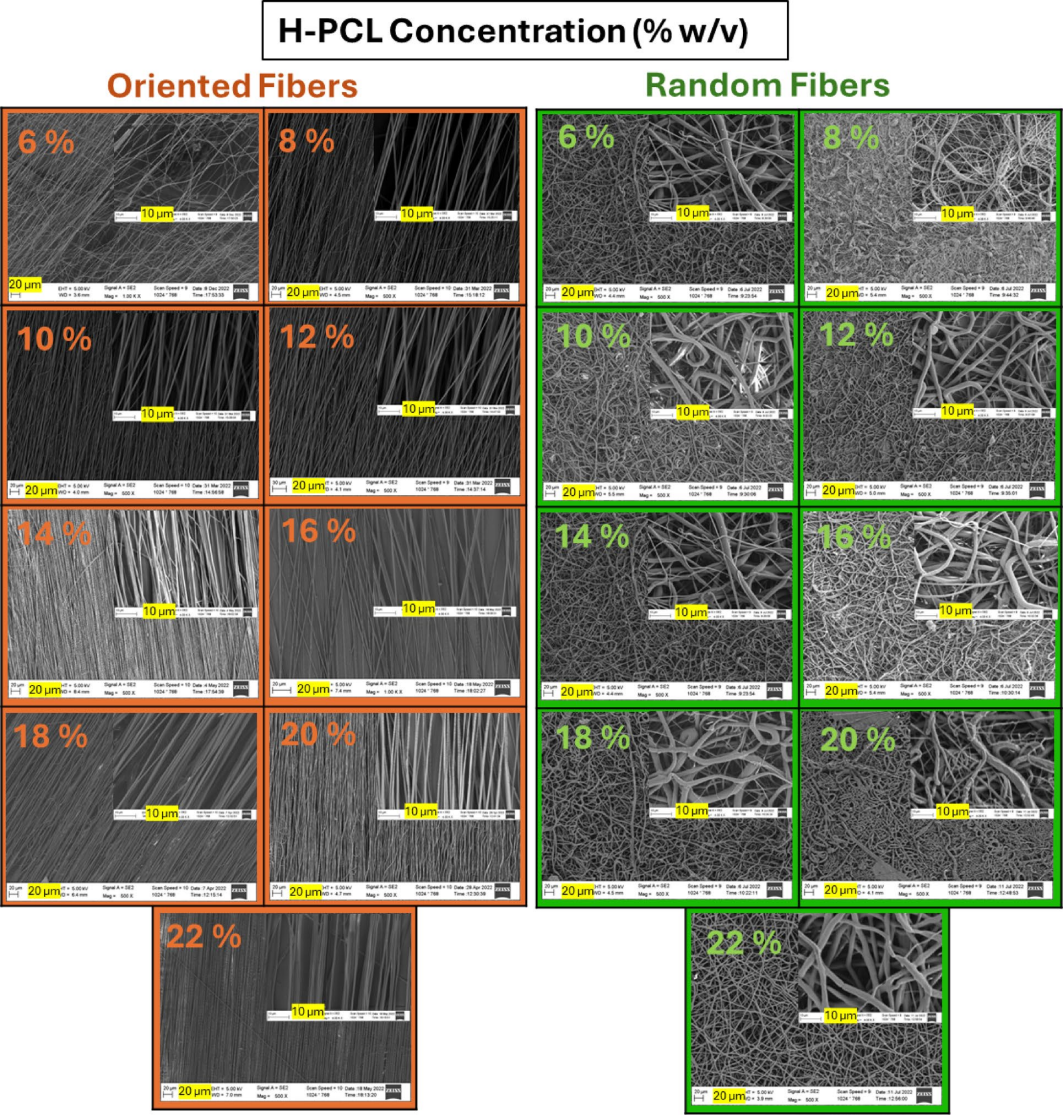
SEM images of electrospun H-PCL fibers prepared at concentrations of 6–22% (w/v) for oriented (left) and random (right) configurations. Scale bars: 20 μm (main images) and 2 μm (insets).

SEM observations (Fig. 1) confirmed that oriented fibers could be reliably produced only within a relatively narrow concentration range. At 6% (w/v), no stable oriented fibers were obtained; instead, deposition consisted of sparse or discontinuous filaments, indicating insufficient chain entanglement to sustain a continuous electrospinning jet. Continuous, well-aligned fibers first appeared at 8% (w/v) and remained stable up to 22% (w/v), where uniform morphology and consistent alignment were maintained.

Random fibers exhibited a broader concentration tolerance. At 6% (w/v), intermittent fibers and bead-like structures were observed, indicating unstable jet formation. At 8% (w/v), discontinuous but recognizable fibers formed, whereas concentrations ≥ 10% (w/v) yielded continuous, defect-free fibrous networks. Across the 10–22% (w/v) range, random fibers displayed progressively thicker and more densely packed morphologies while retaining structural continuity.

At very high concentration (24% w/v), both fiber orientations showed clear signs of excessive viscosity, including polymer accumulation at the needle tip and unstable jet behavior, which prevented reliable fiber formation under the selected processing conditions.

Overall, these results demonstrate that polymer concentration critically governs electrospinnability through its control of viscoelastic stability and chain entanglement density. While both fiber types require sufficient concentration to sustain a continuous jet, oriented fibers demand a higher degree of molecular entanglement and therefore exhibit a narrower processing window. A complete summary of morphological observations is provided in Table 2.

**Table 2:** Optimization of H-PCL solution concentration (%, w/v relative to total solution volume) for electrospinning oriented and random fibers, based on SEM morphological assessment.

H-PCL Concentration (% w/v)	Oriented Fibers – Observation	Random Fibers – Observation
6%	No stable fiber formation (discontinuous filaments)	Intermittent fiber formation; irregular thin fibers
8%	Intermittent fiber formation	Intermittent fiber formation
10%	Continuous, stable fiber formation	Continuous, stable fiber formation
12%	Continuous fibers	Continuous fibers
14%	Continuous fibers	Continuous fibers
16%	Continuous fibers	Continuous fibers
18%	Continuous fibers	Continuous fibers
20%	Continuous fibers	Continuous fibers
22%	Continuous fibers	Continuous fibers
24%	Polymer accumulation at needle tip (jet instability)	Polymer accumulation at needle tip (jet instability)

### Molecular weight blend study

To evaluate the influence of molecular weight distribution on electrospinning behavior, blends of high–molecular-weight PCL (H-PCL, 80 kDa) and low–molecular-weight PCL (L-PCL, 10 kDa) were prepared at a fixed total polymer concentration of 18% (w/v), with the H-PCL fraction varied from 100% to 20% (w/w) in 10% increments. This design enabled systematic assessment of jet stability, fiber continuity, and morphology as a function of molecular weight composition.

**Fig. 2:**
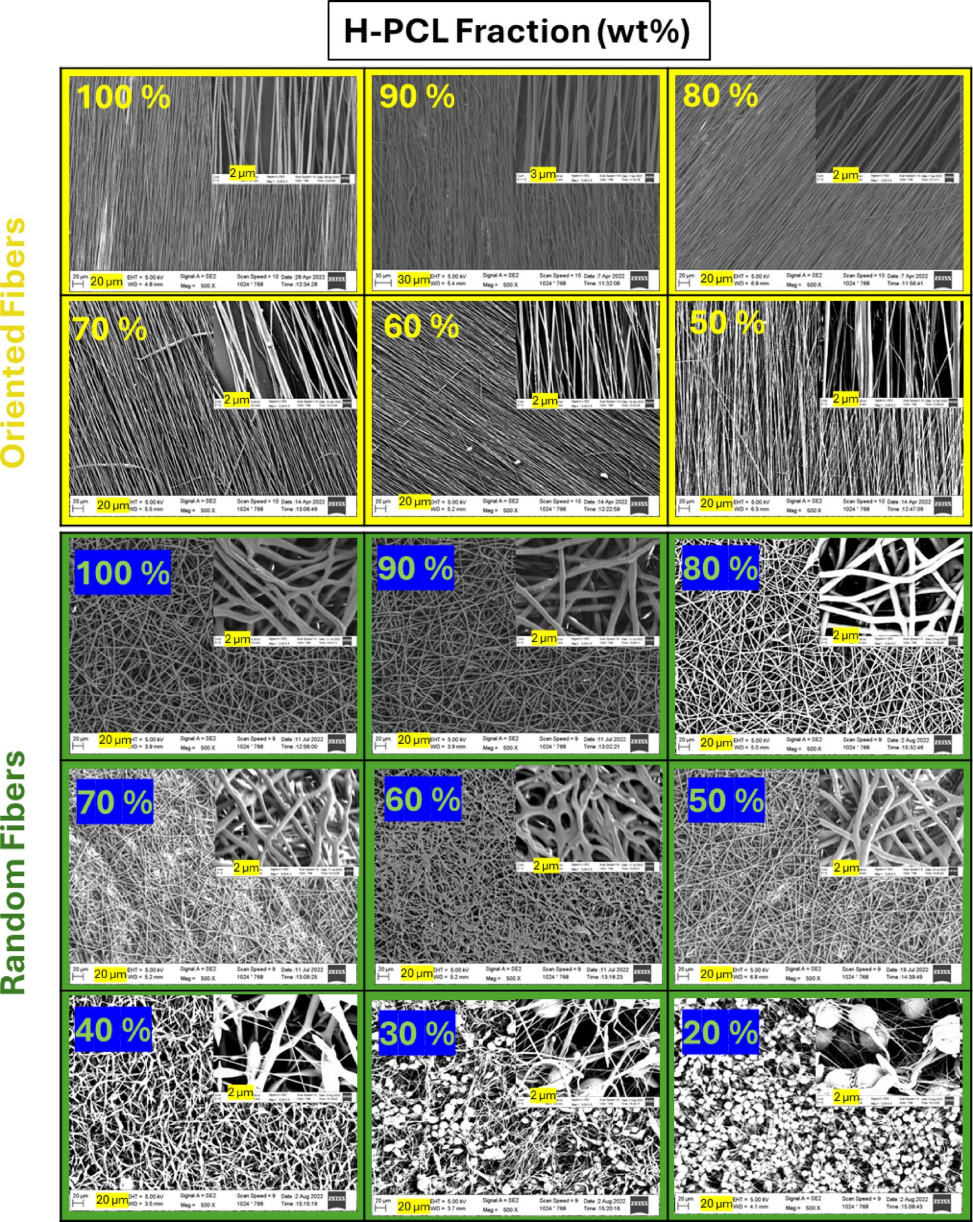
SEM images of electrospun H-PCL/L-PCL fibers prepared at 18% (w/v) with varying H-PCL content. Oriented fibers (100–50% H-PCL) and random fibers (100–20% H-PCL) are shown. Scale bars: 20 μm (main images) and 2 μm (insets).

SEM observations (Fig. 2) showed that oriented fibers could be reliably produced only within a narrower compositional window, specifically 50–100% H-PCL, where continuous and well-aligned fibers were obtained. Below 50%, oriented fiber formation ceased due to loss of jet stability. Random fibers displayed greater tolerance to reduced H-PCL content, maintaining continuous fibers between 50% and 100% and showing intermittent, discontinuous fibers at 40%. At 30% and 20% H-PCL, unstable jetting and electrospraying dominated, producing fragmented deposits rather than continuous fibers. The detailed morphological outcomes are summarized in Table 3.

These results parallel the concentration study (Table 2), confirming that sufficient chain entanglement is essential for stable electrospinning. In addition to total polymer concentration, molecular weight distribution critically governs electrospinnability, particularly for defect-free oriented fibers that require higher viscoelastic stability. Consistent with this interpretation, L-PCL alone did not form continuous fibers under identical conditions, demonstrating that low molecular weight alone cannot sustain stable jet formation.

**Table 3:** Morphology-based optimization of H-PCL fraction for electrospinning.

H-PCL Fraction (% w/w)	Oriented Fibers – Observation	Random Fibers – Observation
100%	Continuous, well-aligned fibers	Dense, uniform continuous fiber network
90%	Continuous fibers	Continuous fibers
80% H-PCL	Continuous fibers	Continuous but mild heterogeneity in thickness
70% H-PCL	Continuous fibers	Continuous fibers with localized thick filaments
60% H-PCL	Continuous with minor diameter variation	Continuous fibers with occasional fused junctions
50% H-PCL	Continuous; slightly less uniform alignment	Continuous fibers; mild structural irregularity
40% H-PCL	No fiber formation	Intermittent fibers with bead formation
30% H-PCL	No electrospinning (jet not initiated)	Discontinuous jets and beaded structures
20% H-PCL	No electrospinning (jet not initiated)	Electrosprayed droplets and non-fibrous deposits

### Fabrication of oriented fibers

**Fig. 3:**
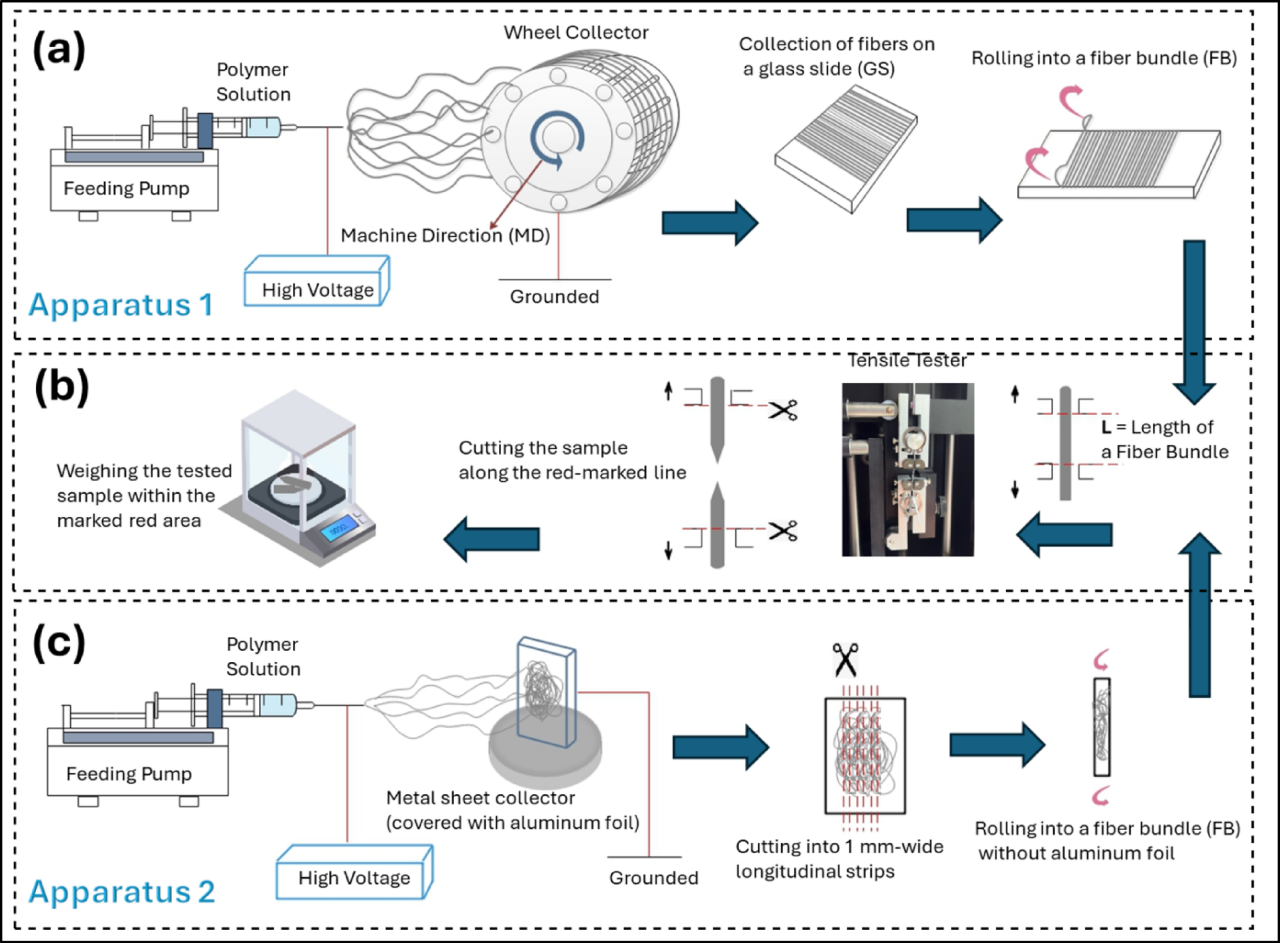
Schematic illustration of the electrospinning and testing workflow. (**a**) Electrospinning apparatus 1 with a rotating drum for fabrication of oriented fibers. (**b**) Electrospinning apparatus 2 with a static flat collector for fabrication of random fibers. (**c**) Tensile testing procedure for both oriented and random fiber bundles.

Oriented PCL fibers were fabricated using electrospinning apparatus 1, equipped with a rotating cylindrical drum collector with two circular terminal plates containing evenly spaced notches (Fig. 3a.). Horizontal metal bars were inserted into the notches at 25 mm spacing to support fiber collection. The drum (diameter 21.2 cm) was rotated at 100 rpm (tangential surface velocity ≈ 1.1 m/s), sufficient to induce preferential fiber orientation while maintaining stable deposition and avoiding fiber breakage. Fiber alignment was confirmed by SEM images in Sect.  2.3 and 2.4 (Figs. 1 and 2).

During electrospinning, PCL jets were elongated under the electric field and deposited onto the rotating collector, resulting in fibers aligned along the machine direction. Aligned fibers were carefully removed from the collector bars, transferred onto glass slides, and assembled into bundles for tensile testing (Fig. 3b).

### Fabrication of random fibers

Randomly oriented PCL fibers were produced using electrospinning apparatus 2 with a static flat metal collector covered with aluminum foil (Fig. 3c). Fibers deposited without imposed motion were collected in a non-aligned configuration. Electrospun mats were cut into 1 mm-wide strips, and the aluminum foil was removed prior to rolling fibers into bundles for tensile testing. The deposition duration was controlled to maintain thin, uniform mats, ensuring precise cutting and reproducible mechanical measurements. The tensile testing procedure for random fibers is illustrated in Fig. 3b.

### Soaking experiments

Electrospun PCL fibers were soaked in acetic acid, formic acid, and phosphate-buffered saline (PBS) to assess their chemical and physiological stability. Acidic media were selected to evaluate the stability of PCL fibers under conditions relevant to biomaterial processing and potential localized biological microenvironments, where mild acidic conditions may occur during tissue remodeling or inflammatory responses. PBS was used as a physiologically relevant buffered medium (pH ~ 7.4) to simulate aqueous conditions commonly employed in cell culture and in vitro degradation studies of biomedical scaffolds. Fibers were exposed under controlled conditions, and subsequent effects on morphology, mechanical integrity, and degradation were evaluated. Temperature-dependent experiments were additionally conducted to compare degradation behavior under laboratory conditions (~ 25 °C) and physiologically relevant conditions (37 °C), corresponding to typical cell culture and in vivo environments. Detailed procedures for each medium are described below.

#### Acidic media

Electrospun PCL fibers fabricated from 18% (w/v) H-PCL solutions were subjected to solvent-soaking treatments to evaluate the influence of acidic environments on their mechanical behavior. Fiber bundles were immersed separately in formic acid/water (FA/H₂O) and acetic acid/water (AA/H₂O) binary solutions, with acid concentrations ranging from 0% to 100% (v/v) in 5% increments. All immersions were conducted at room temperature (≈ 25 °C) for 24 h to allow adequate solvent penetration and polymer–solvent interaction.

**Fig. 4:**
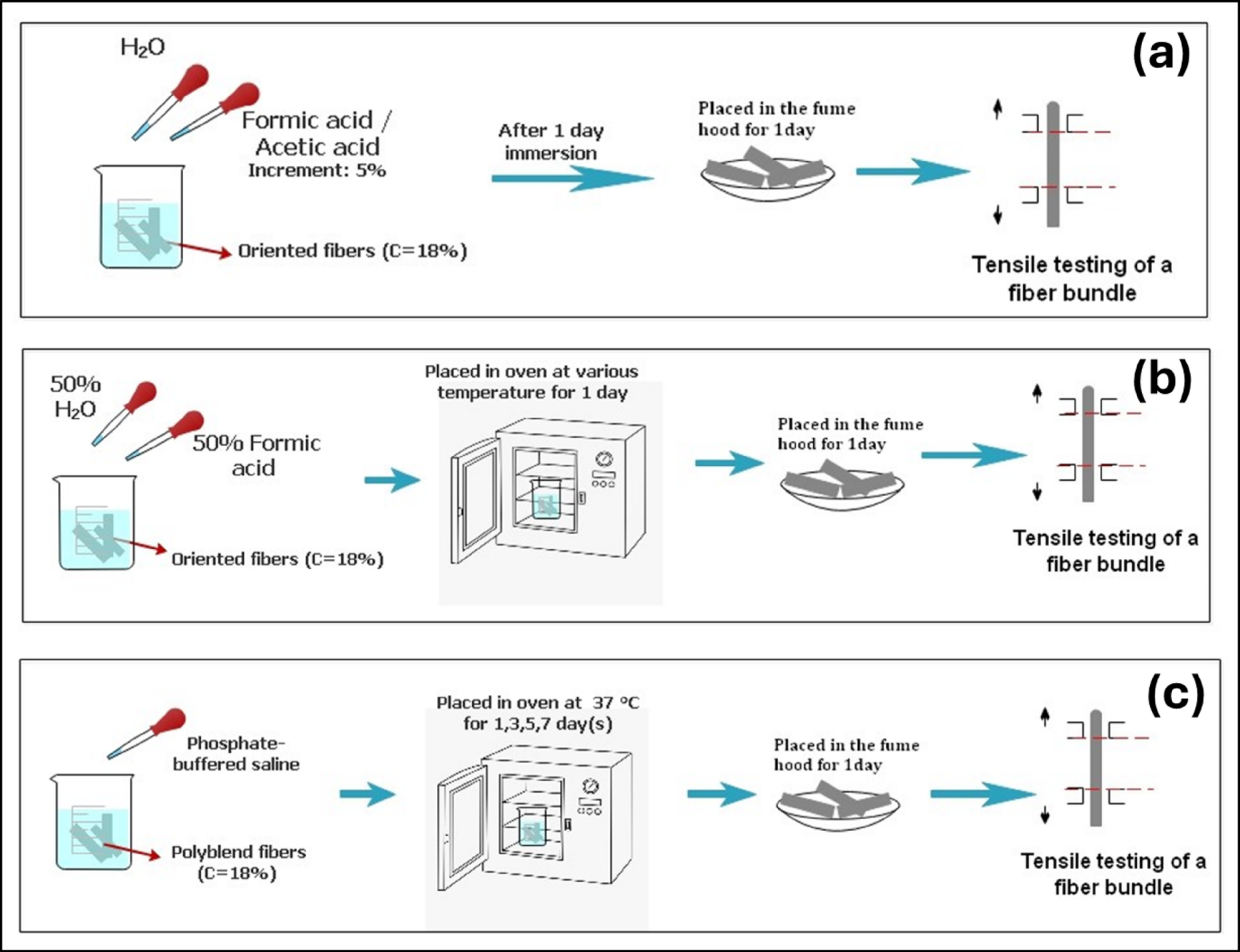
Schematic illustration of the solvent-soaking treatments applied to electrospun PCL fibers: (**a**) immersion in FA/H₂O and AA/H₂O solutions with varying concentrations; (**b**) temperature-dependent soaking in FA/H₂O; and (**c**) soaking of H-PCL/L-PCL blend fibers in PBS to evaluate physiological conditions.

After soaking, the samples were removed, carefully straightened, and placed on clean Petri dishes. The fibers were then dried in a fume hood for 24 h to ensure complete removal of residual solvent and eliminate plasticization effects prior to mechanical evaluation. The overall procedure is illustrated schematically in Fig. 4a.

#### Temperature-dependent acidic media

To assess the role of temperature on solvent-induced modifications, additional H-PCL fiber bundles were immersed in 50% (v/v) FA/H₂O and incubated at controlled temperatures between 20 °C and 40 °C in 5 °C intervals using a laboratory oven. This study enabled evaluation of thermal effects on polymer swelling, microstructural relaxation, and resulting mechanical properties.

After soaking for 24 h, the samples were removed, transferred to Petri dishes, and dried for 24 h in a fume hood to ensure complete solvent evaporation. The corresponding experimental workflow is presented in Fig. 4b.

#### PBS soaking

To examine the influence of aqueous physiological conditions, both oriented and random fibers obtained from H-PCL/L-PCL blend solutions were immersed in phosphate-buffered saline (PBS) and incubated at 37 °C for durations ranging from 1 to 7 days. After each soaking period, fibers were removed from the PBS solution, dried for 24 h in a fume hood, and processed for mechanical testing. The procedural schematic is shown in Fig. 4c.

### Fiber diameter measurements

The diameters of electrospun PCL nanofibers were determined using scanning electron microscopy (SEM). Oriented fibers were collected directly onto glass slides from the rotating cylinder collector, ensuring that fiber alignment was preserved during transfer. Random fibers were prepared by cutting small sections of the aluminum foil–supported fiber mats. All samples were mounted on SEM stubs and sputter-coated with a thin gold layer using a Q150T S coater (Quantum Design Europe; WW5 Gold 1 program) to minimize charging.

SEM imaging was performed using an AURIGA-4750 microscope (ZEISS, Germany). Low-magnification images (500× and 3000×) were acquired to examine overall fiber morphology and uniformity, while high-magnification images (5000×) were used for precise diameter measurements. For each sample, 50 measurement points were selected manually and randomly across the fiber matrix to ensure unbiased sampling. Fiber diameters were measured using ImageJ, and statistical parameters including mean and median values were calculated using Origin software. This procedure provided reproducible and representative assessments of nanofiber diameter distributions for all oriented and random samples^43,45^.

### Mechanical testing

The mechanical behavior of electrospun polycaprolactone (PCL) fibers was characterized using a Vibrodyn 400 tensile tester (Lenzing Instruments, Austria). Electrospun fibers were carefully collected and rolled into compact fiber bundles to ensure a cohesive and reproducible mechanical response. The resulting fiber mats were cut into strips 1 mm in width and 20 mm in length, and all tensile tests were performed using a fixed gauge length, *L*, of 5 mm. The schematic of the tensile testing procedure for oriented and random fibers is shown in Fig. 3b.

Tensile measurements were conducted at room temperature under a preload of 100 mg and a constant crosshead speed of 50 mm·min⁻¹. For each fiber type, three independent specimens were tested. Following tensile failure, the fractured portion of each fiber bundle within the gauge region was collected and weighed to determine the bundle mass, *W*, using an analytical balance. The polymer density, *ρ*, was taken from supplier specifications^20,21^.

Force–strain data were recorded continuously during testing. Smooth and continuous curves confirmed that the fiber bundles behaved as mechanically integrated structures, without evidence of progressive pull-out or failure of individual fibers. From each force–strain curve, the force at break, *F*_*max*_, the strain at break, *ε*_*max*_, and the slope of the elastic region, *k*, were extracted. The slope *k* was consistently determined in the quasi-linear elastic region at approximately 25% strain to minimize the influence of viscoelastic effects and initial toe-region artifacts.

Mechanical properties were calculated under the assumption that each fiber bundle behaves as an ideal cylindrical specimen with a uniform effective cross-sectional area^45^. The total volume of the fiber bundle within the gauge length, *V*, was calculated from its mass and density:

1 $$\:V=\frac{W}{\rho\:}$$

The effective cross-sectional area of the fiber bundle, *A*, was then obtained by dividing the volume by the gauge length:

2 $$\:A=\frac{V}{L}$$

The engineering stress, *σ*, was calculated from the applied force, *F*, and the effective cross-sectional area:

3 $$\:\sigma\:=\frac{F}{A}$$

Young’s modulus, *E*, was determined from the ratio of stress to strain, *ε*, within the elastic region:

4 $$\:E=\frac{\sigma\:}{\epsilon\:}$$

Similarly, the tensile strength, *σ*_*max*_, was calculated using the maximum force at break:

5 $$\:{\sigma\:}_{\mathrm{m}\mathrm{a}\mathrm{x}}=\frac{{F}_{\mathrm{m}\mathrm{a}\mathrm{x}}}{A}$$

By combining Eqs. (1)–(5), Young’s modulus, Eq. (6), and the tensile strength Eq. (7), were directly expressed in terms of experimentally measured parameters (bundle stiffness *k*, force at break *F*_*max*_, bundle mass *W*, gauge length *L*, and polymer density *ρ*:

6 $$\:E=k\cdot\:\frac{\rho\:L}{W}$$

7 $$\:{\sigma\:}_{\mathrm{m}\mathrm{a}\mathrm{x}}={F}_{\mathrm{m}\mathrm{a}\mathrm{x}}\cdot\:\frac{\rho\:L}{W}$$

This approach enabled consistent and reproducible quantification of Young’s modulus, tensile strength, force at break, and strain at break for both oriented and random electrospun PCL fiber bundles. Using specimen dimensions and the PCL density (≈ 1.145 g cm⁻³), the fiber volume fraction in each bundle was estimated from its mass, so the reported Young’s modulus reflects the effective modulus of the porous scaffold rather than a fully dense material^20,21^.

## Results and discussion

In the Results and Discussion, the effects of polymer concentration, molecular-weight blending, acid-induced degradation, and phosphate-buffered saline (PBS)-induced degradation on the mechanical properties of electrospun PCL fibers are systematically presented and thoroughly analyzed.

### Effects of polymer concentration

**Fig. 5:**
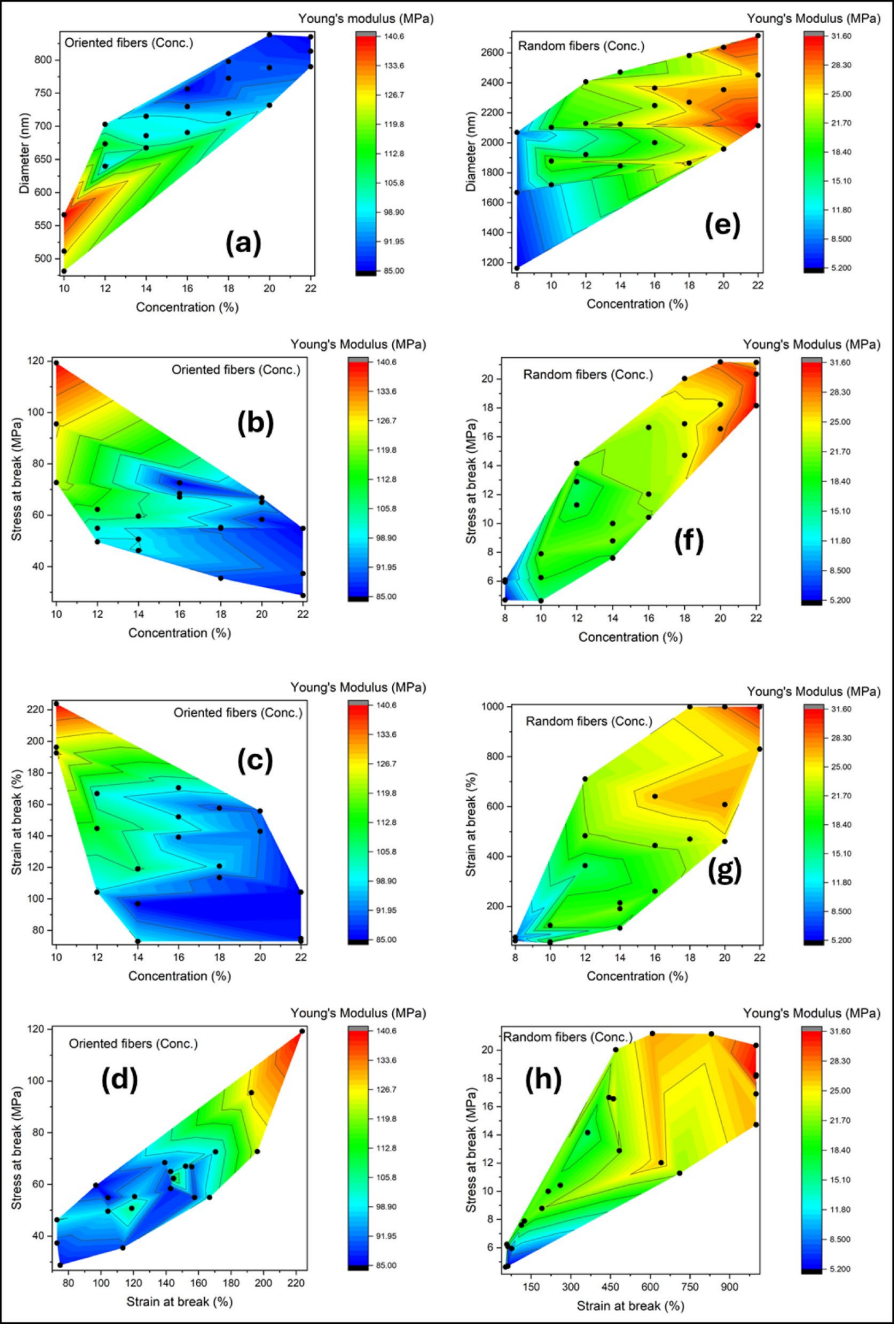
Contour plots of Young’s modulus (MPa) of electrospun PCL fibers as a function of polymer concentration, fiber diameter, stress at break, and strain at break. (**a**–**d**) Oriented fibers; (**e**–**h**) random fibers. Panels show: (**a**,** e**) diameter vs. concentration; (**b**,** f**) stress at break vs. concentration; (**c**,** g**) strain at break vs. concentration; and (**d**,** h**) stress vs. strain at break. Black dots represent experimental data.

The mechanical properties of PCL electrospun fibers were systematically analyzed as functions of polymer concentration, fiber diameter, stress at break, and strain at break. The study compared oriented and random fibers, presenting results through eight contour plots (Fig. 5a–h) that illustrate variations in Young’s modulus (MPa) across these parameters. In all plots, warmer colors (red) correspond to higher modulus values, while cooler colors (blue) indicate lower stiffness. Experimental data points are represented by black dots, and the contour surfaces were interpolated from these measurements.

The effect of fiber orientation on Young’s modulus is immediately apparent. In oriented fibers (plots a–d), the highest modulus (~ 140 MPa) is observed at low polymer concentrations (10–12%) and small fiber diameters (~ 550–600 nm). This is likely due to enhanced chain alignment along the fiber axis, which facilitates load transfer and reduces structural defects, as reported in previous studies^46,47^. As concentration and fiber diameter increase, modulus decreases, reaching lower values at high concentrations (~ 20–22%) and larger diameters (~ 750–850 nm). In contrast, random fibers (plots e–h) exhibit substantially lower maximum modulus (~ 30 MPa) across a broader range of diameters (1200–2600 nm). For random fibers, the gradual increase in modulus with concentration may be attributed to higher polymer chain entanglement, but overall stiffness remains limited due to the absence of alignment. These observations are consistent with literature showing that fiber orientation is a dominant factor in enhancing stiffness^48^.

The relationship between stress at break, polymer concentration, and modulus further underscores the distinction between oriented and random fibers. Oriented fibers reach maximum modulus at low concentrations and high stress at break (~ 120 MPa), highlighting that alignment enables efficient load transfer along the fiber axis. Modulus decreases with increasing concentration, consistent with the observed diameter-dependent trends. Random fibers, however, require both high concentration and high stress at break (~ 20 MPa) to reach their modest maximum modulus (~ 30 MPa), reflecting their weaker load-bearing capability due to random orientation and larger fiber diameters.

Strain at break reveals a complementary pattern. Oriented fibers attain high stiffness at relatively low strains (~ 100–120%), while high strain values (> 160%) correspond to regions of low modulus. Random fibers, in contrast, can accommodate much larger strains (up to 1000%) but exhibit correspondingly lower modulus values. This indicates that the flexibility of random fibers comes at the expense of stiffness, demonstrating a clear trade-off between extensibility and mechanical strength. Such trade-offs are well-documented in electrospun polymer systems^46^. Stress–strain contour plots further confirm this behavior: oriented fibers are strong and stiff but moderately extensible, whereas random fibers are softer and highly ductile, with lower stress and modulus values.

Overall, several key trends emerge from these analyses. Fiber orientation dramatically enhances Young’s modulus, stress at break, and overall stiffness, whereas random fibers primarily confer high extensibility. Fiber diameter affects the modulus, although the relationship depends on processing conditions; smaller diameters are associated with higher stiffness in certain regions, particularly for oriented fibers. Polymer concentration modulates these effects differently depending on orientation: oriented fibers achieve maximum stiffness at lower concentrations, while random fibers require higher concentrations to optimize modulus. These findings are consistent with previous reports on electrospun PCL fibers^46–48^, and provide further insight into how processing parameters can be tuned to balance stiffness and flexibility.

### Effect of molecular weight blending

**Fig. 6:**
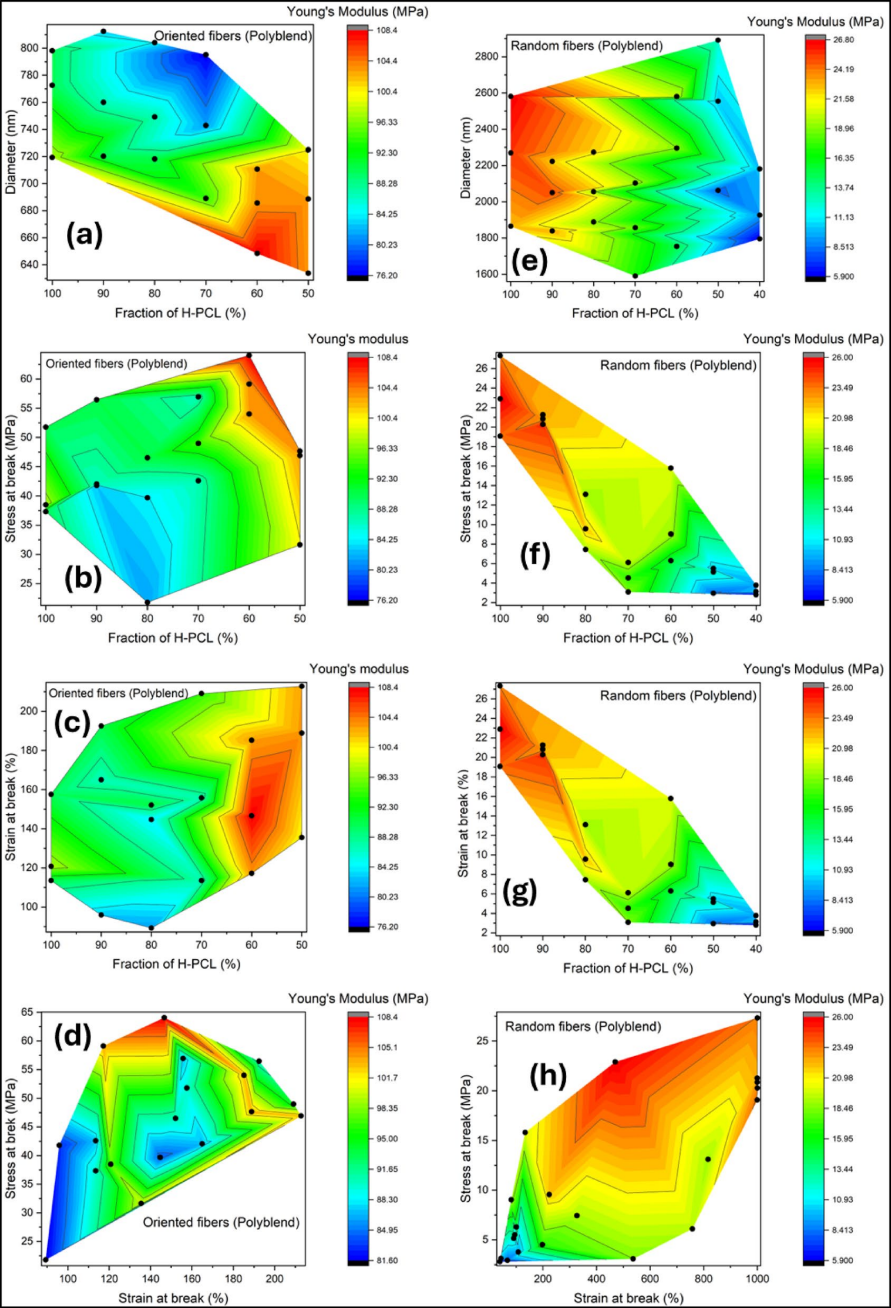
Contour plots of Young’s modulus (MPa) of electrospun PCL fibers as a function of H-PCL fraction, fiber diameter, stress at break, and strain at break. (**a**–**d**) Oriented fibers; (**e**–**h**) random fibers. Panels show: (**a**,** e**) diameter vs. H-PCL fraction; (**b**,** f**) stress at break vs. H-PCL fraction; (**c**,** g**) strain at break vs. H-PCL fraction; and (**d**,** h**) stress vs. strain at break. Black dots represent experimental data.

Figure 6 presents a comprehensive analysis of the mechanical behavior of PCL electrospun fibers prepared from polyblend solutions containing high-molar-mass PCL (H-PCL) and low-molar-mass PCL (L-PCL). In Fig. 6, eight contour plots (a–h) illustrate variations in Young’s modulus (MPa) as a function of polymer fraction, fiber diameter, stress at break, and strain at break. Experimental measurements are denoted by black dots, while the colored contours represent interpolated surfaces. Warmer colors (red) indicate higher modulus values, corresponding to stiffer fibers, whereas cooler colors (blue) indicate lower stiffness.

The relationship between fiber diameter, H-PCL fraction, and Young’s modulus reveals distinct behaviors for oriented and random fibers. In oriented fibers (plot a), the modulus ranges from approximately 76 to 108 MPa, with maximum stiffness observed at low H-PCL fractions (~ 50–60%) and smaller fiber diameters (~ 650–700 nm). This trend suggests that fibers with higher L-PCL content and thinner diameters allow better chain alignment and reduced defect density, enhancing load transfer along the fiber axis^49^. As fiber diameter increases or H-PCL content rises, the modulus gradually decreases, reflecting reduced stiffness. In contrast, random fibers (plot e) exhibit much lower modulus values, ranging from ~ 6 to 27 MPa. Maximum stiffness in these fibers occurs at moderate diameters (~ 2000–2600 nm) and high H-PCL fractions (~ 80–100%), indicating that random fibers rely more on molecular weight content than alignment to achieve mechanical performance, though they remain weaker than oriented fibers. This behavior aligns with previous reports on random PCL fibers^50^.

Analysis of stress at break versus H-PCL fraction further emphasizes the role of alignment in mechanical performance. Oriented fibers (plot b) achieve a maximum modulus of ~ 108 MPa at high stress at break (~ 60 MPa) and low H-PCL fractions (~ 50–60%). This demonstrates that aligned fibers with higher L-PCL content efficiently transfer load and maintain stiffness under moderate stress. Conversely, random fibers (plot f) exhibit lower modulus (~ 6–26 MPa), with maximum stiffness observed at high H-PCL fractions (~ 90–100%) and stress levels of ~ 20–25 MPa. These results confirm that fiber alignment is critical for load transfer in oriented fibers, whereas random fibers require both high molecular weight content and stress to achieve moderate stiffness.

The influence of strain at break on Young’s modulus also highlights a trade-off between stiffness and extensibility. Oriented fibers (plot c) display maximum modulus at low-to-moderate strains (~ 120–160%) and low H-PCL fractions (~ 50–60%). At higher strains (> 180%), the modulus decreases, indicating that these fibers are stiff but moderately extensible. Random fibers (plot g) can accommodate substantially larger strains, up to ~ 1000%, with maximum modulus (~ 26 MPa) observed at high strains (~ 600–800%) and high H-PCL content. This demonstrates that while oriented fibers are optimal for load-bearing applications due to high stiffness, random fibers offer superior ductility for flexible or stretchable materials^51^.

Stress versus strain at break further corroborates these trends. Oriented fibers (plot d) exhibit the highest modulus at high stress (~ 60 MPa) and moderate strain (~ 120%), reinforcing the notion that fiber alignment enhances stiffness and strength but limits extensibility. Random fibers (plot h) show a gradual increase in modulus with increasing stress and strain, reaching a maximum of ~ 26 MPa at high stress (~ 20 MPa) and high strain (~ 700–900%). These observations confirm that random fibers are softer and more ductile, whereas oriented fibers combine higher stiffness with moderate extensibility^52,53^.

Overall, the data highlight several key trends. Fiber orientation consistently governs mechanical performance, with oriented fibers achieving higher modulus due to enhanced load transfer along the fiber axis. H-PCL fraction modulates stiffness differently depending on fiber alignment: oriented fibers reach maximum modulus at lower H-PCL fractions, whereas random fibers require higher H-PCL content for moderate stiffness. Fiber diameter also influences mechanical properties, with smaller diameters promoting higher stiffness in oriented fibers, whereas diameter effects are less pronounced in random fibers. These effects are likely due to improved molecular chain alignment and reduced defects in thinner fibers formed under stronger extensional forces during electrospinning. Finally, a clear trade-off exists between stiffness and extensibility: oriented fibers exhibit higher modulus and are therefore better suited for load-bearing applications, while random fibers, which accommodate larger deformations, are more appropriate for flexible and stretchable materials^52–54^.

### Combined mechanical trends

**Fig. 7:**
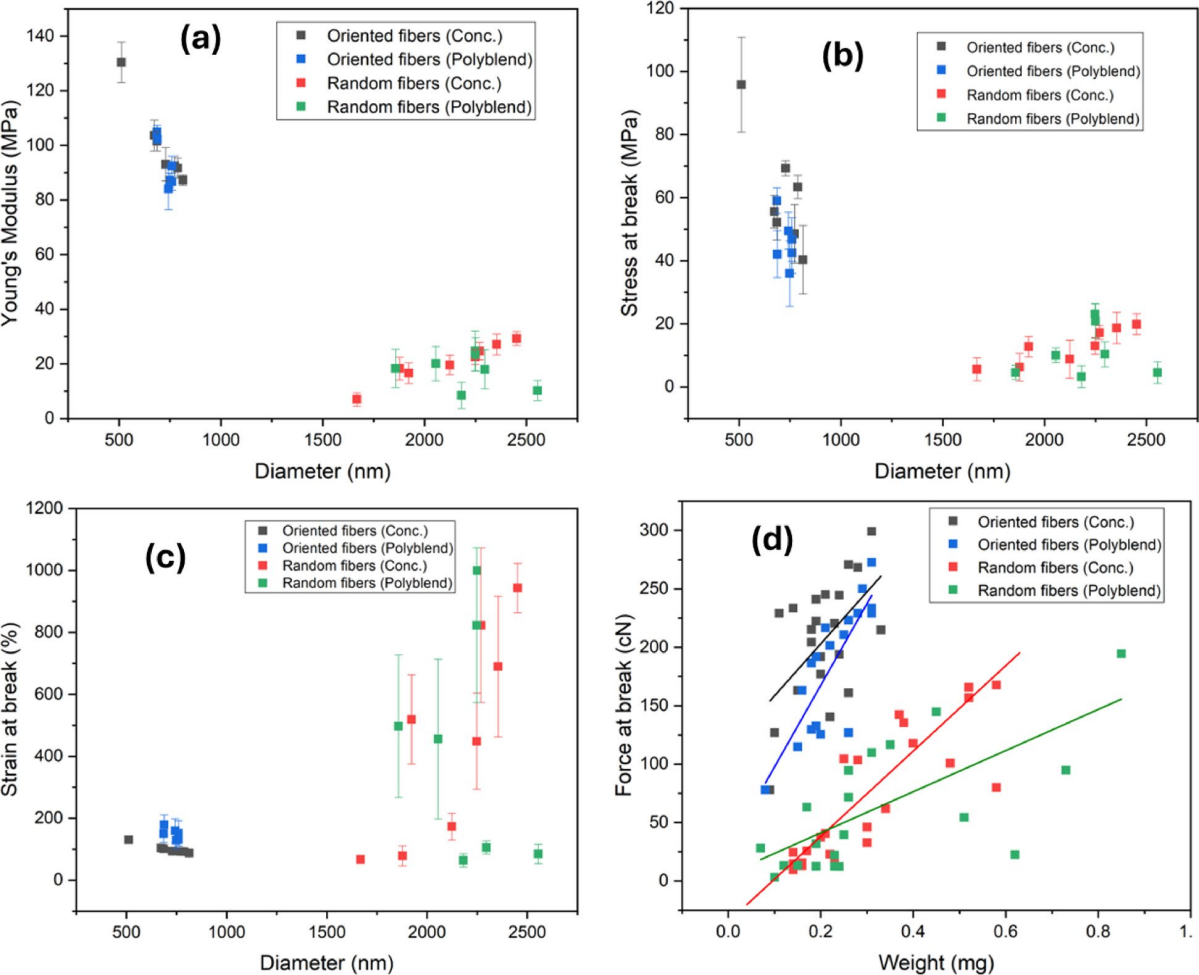
Mechanical properties of oriented and random electrospun PCL fibers as a function of fiber diameter and sample weight, including samples prepared with varying polymer concentration (Conc.) and H-PCL fraction (polyblend). (**a**) Young’s modulus vs. diameter; (**b**) stress at break vs. diameter; (**c**) strain at break vs. diameter; and (**d**) force at break vs. weight.

Figure 7 presents a comparative assessment of the mechanical behavior of oriented and random PCL fibers produced from polymer concentration solutions (Conc.) and polyblends of high- and low-molar-mass PCL (H-PCL/L-PCL). The panels illustrate Young’s modulus, stress at break, strain at break, and force at break as functions of fiber diameter or sample weight, highlighting the combined effects of fiber orientation, polymer concentration, and molecular weight blending.

Panel (a) shows Young’s modulus versus diameter. Oriented fibers, regardless of Conc. or polyblend composition, display markedly higher modulus values (~ 90–130 MPa) at smaller diameters (~ 500–800 nm) compared to random fibers (~ 7–30 MPa) with larger diameters (~ 1700–2500 nm). Random fibers exhibit greater variability, whereas oriented polyblend fibers show slightly reduced but comparable stiffness to their Conc. counterparts. These trends indicate that fiber alignment strongly enhances stiffness, likely due to improved chain orientation and more efficient load transfer along the fiber axis. The superior modulus in smaller-diameter oriented fibers suggests enhanced molecular packing, while the slight reductions in polyblend fibers may arise from phase heterogeneity and imperfect molecular interactions^55,56^.

Panel (b) presents stress at break versus diameter. Oriented fibers again outperform random fibers, sustaining stresses of 40–100 MPa compared to 5–25 MPa. Stress decreases with increasing diameter for oriented fibers, reflecting reduced structural efficiency at larger scales. Random fibers, irrespective of composition, cluster at low stress values with substantial scatter, consistent with their disordered morphology. Polyblend-oriented fibers follow a similar trend but with marginally lower stress, suggesting that blending slightly compromises maximum tensile strength.

Panel (c) depicts strain at break versus diameter. Random fibers exhibit exceptionally high extensibility (up to ~ 1000%), while oriented fibers show limited strain (~ 70–150%), demonstrating the trade-off between stiffness and flexibility. Polyblend random fibers display moderately higher strain than Conc. random fibers, indicating that blending increases chain mobility and ductility. These results confirm that fiber alignment governs the balance between stiffness and extensibility.

Panel (d) shows force at break versus sample weight. Oriented fibers achieve the highest breaking forces (up to ~ 280 cN), reflecting their elevated modulus and tensile strength, whereas random fibers produce lower forces despite larger diameters. The steeper slopes for oriented fibers indicate more effective load bearing with increasing weight. Random fibers show weaker scaling due to misaligned polymer chains and less efficient load transfer.

Collectively, these results underscore fiber orientation as a dominant factor controlling mechanical performance, with oriented fibers exhibiting high stiffness and strength but reduced extensibility, while random fibers provide greater flexibility at the expense of mechanical robustness. Concentration-controlled fibers generally outperform polyblend fibers in stiffness and strength, whereas polyblend fibers enhance extensibility, particularly in random configurations. The consistently larger diameters observed for random fibers approximately threefold higher than those of oriented fibers are attributed to differences in electrospinning dynamics rather than measurement artifacts. Specifically, oriented fiber collection imposes additional mechanical drawing and elongational strain during deposition, promoting jet stretching and thinning, whereas random fiber collection lacks this secondary drawing step, resulting in thicker fibers. Consequently, diameter effects are pronounced in oriented fibers but less impactful in random fibers.

Together, fiber orientation, diameter, polymer concentration, and molecular-weight blending dictate tunable mechanical behavior in electrospun PCL fibers, providing guidance for designing materials for load-bearing or flexible biomedical applications^57–60^.

### Effect of acid-induced degradation

**Fig. 8:**
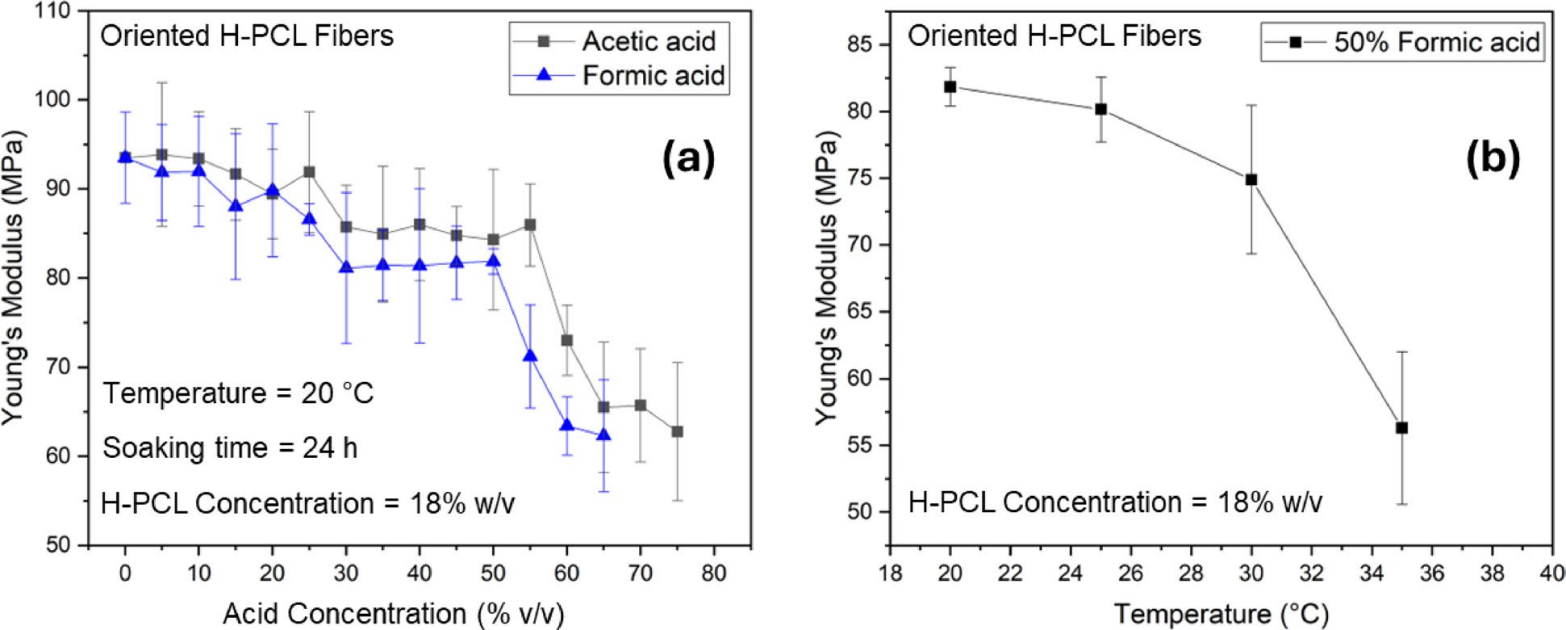
Young’s modulus of oriented H-PCL fibers (18% w/v) after solvent soaking treatments. (**a**) Fibers soaked in acetic acid/water (AA/H₂O) and formic acid/water (FA/H₂O) mixtures with varying acid concentrations. (**b**) Fibers soaked in 50% FA/H₂O at different temperatures.

Figure 8a illustrates the degradation behavior and corresponding mechanical response of oriented H-PCL fibers (fabricated from an 18% w/v H-PCL solution) after soaking in increasing concentrations of acetic acid/water (AA/H₂O) and formic acid/water (FA/H₂O) mixtures, ranging from 0% to 100% pure acid. These acids were chosen as representative, biomedically relevant reagents due to their widespread use in polymer processing and surface modification, their relatively mild acidity compared to strong mineral acids, and their compatibility with biomedical materials. Moreover, acetic and formic acids provide distinct degradation strengths and diffusion characteristics, allowing controlled hydrolytic chain scission and enabling systematic comparison of acid-induced effects. Fibers were immersed for 24 h, followed by complete drying prior to tensile testing.

A clear reduction in Young’s modulus is observed with increasing acid concentration for both AA and FA systems. At low to moderate concentrations, the modulus decreases gradually, likely due to partial hydrolysis and surface erosion. However, beyond a threshold, mechanical degradation accelerates sharply. In AA/H₂O solutions, significant structural shrinkage occurs at ~ 70% acid, rendering the fibers too short and brittle for mechanical testing. At 75% AA, the fibers fully dissolve. Across the measurable range, Young’s modulus decreases from approximately 90 MPa to ~ 60 MPa. These trends indicate that acid concentration directly controls the extent of hydrolytic degradation, consistent with prior studies on PCL hydrolysis^61^.

A similar trend is noted for FA/H₂O, but degradation occurs at lower concentrations. The fibers exhibit pronounced shrinkage at 65% FA and completely dissolve at 70% FA. This confirms that FA induces faster hydrolysis compared to AA due to its stronger acidity and higher solvent power toward PCL chains. The measurable mechanical reduction again spans from ~ 90 MPa to ~ 60 MPa. Concentrations beyond these limits are not reported, as no intact samples remained for testing.

Figure 8b presents the effect of temperature on the stability of oriented 18% H-PCL fibers soaked in 50% FA/H₂O for 24 h. After evaporation of residual solvent, tensile properties were obtained from the remaining fibers. The results show that increasing temperature accelerates acid-induced degradation, as higher thermal energy enhances polymer chain mobility and hydrolysis kinetics. The fibers begin to exhibit shrinkage at 35 °C, and complete dissolution occurs at 40 °C. Within the range where testing was possible, Young’s modulus decreases from approximately 80 MPa to ~ 50 MPa, demonstrating the combined thermo-chemical sensitivity of PCL in acidic environments. These observations align with prior reports highlighting temperature-dependent hydrolysis in biodegradable polymers^62^.

### Effect of PBS-buffered solution–induced degradation

The degradation behavior of polyblend H-PCL/L-PCL electrospun fibers was evaluated by soaking the samples in phosphate-buffered saline (PBS, pH 7.4) at 37 °C for up to 7 days, mimicking physiologically relevant aqueous conditions commonly used to assess the in vitro stability of biodegradable polymeric materials. PBS was selected as a standardized, biocompatible medium that maintains constant ionic strength and pH, enabling reproducible evaluation of hydrolytic degradation and its impact on mechanical performance under conditions relevant to biomedical applications.

**Fig. 9:**
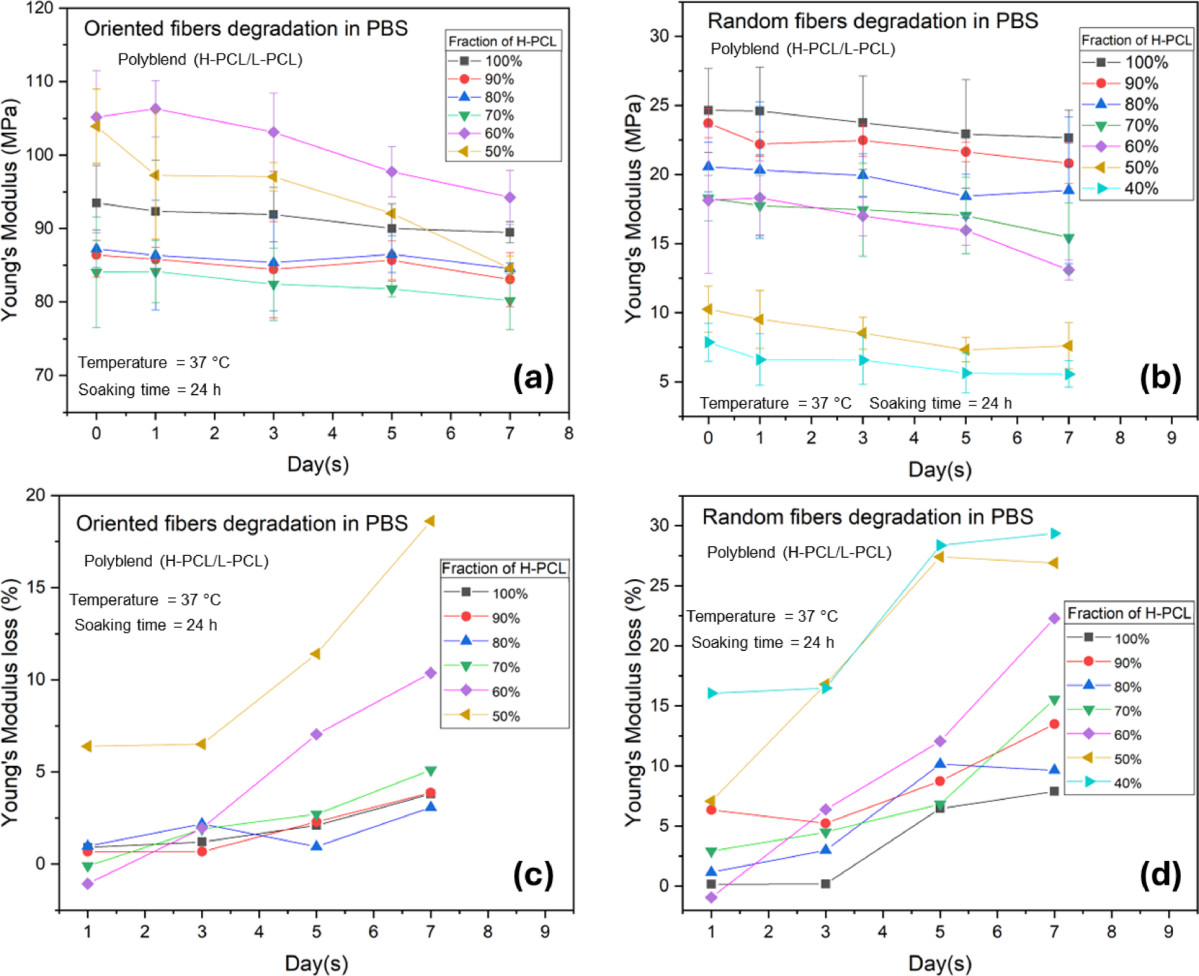
Mechanical properties of oriented and random H-PCL/L-PCL polymer blend fibers in PBS over seven days: (**a**) Young’s modulus of oriented fibers. (**b**) Young’s modulus of random fibers. (**c**) Percentage loss in Young’s modulus of oriented fibers from panel (**a**). (**d**) Percentage loss in Young’s modulus of random fibers from panel (**b**).

Figure 9a and b show the evolution of Young’s modulus over time for oriented and random fibers, respectively, prepared with varying fractions of high-molar-mass PCL (H-PCL). For oriented fibers, the H-PCL fraction ranged from 100% to 50%, whereas for random fibers, the H-PCL fraction ranged from 100% to 40%.

Overall, polyblend fibers exhibited greater stability during PBS exposure than in acidic environments, particularly at moderate to high acid concentrations (≥ 15% v/v) of acetic acid or formic acid. At lower acid concentrations, the extent of mechanical degradation was less pronounced and more comparable to that observed in PBS, highlighting the concentration-dependent nature of hydrolytic destabilization. It should be noted that PBS, a buffered aqueous medium at physiological pH (7.4), is not directly comparable to acid solutions on a concentration basis; instead, it serves as a neutral reference environment representative of physiological conditions.

Within PBS, pure H-PCL (100%) demonstrated the highest stability in both oriented and random configurations, with only minor reductions in stiffness over the 7-day period. As the proportion of H-PCL decreased, the rate and magnitude of mechanical degradation increased, reflecting the higher susceptibility of lower-molecular-weight L-PCL to hydrolytic chain scission under aqueous conditions. Consequently, polyblend fibers with reduced H-PCL content showed more pronounced declines in Young’s modulus during PBS exposure.

Figure 9c and d summarize these trends by presenting the percentage loss in Young’s modulus. The most substantial modulus reductions occurred in blends with the lowest H-PCL content. For oriented fibers, the 50% H-PCL blend showed a modulus loss of approximately 6% after one day and ~ 20% after seven days. For random fibers, the 40% H-PCL composition exhibited even greater losses, decreasing by ~ 15% on day 1 and ~ 30% by day 7. These observations indicate that random fibers are slightly more sensitive to PBS-induced degradation than oriented fibers, likely due to less efficient molecular alignment and load distribution along the fiber axis.

Collectively, these data demonstrate that the degradation resistance of PCL polyblend fibers in physiological buffer is strongly governed by both the molecular-weight composition and fiber orientation. Higher H-PCL content and fiber alignment contribute to improved stability, whereas increasing contributions from L-PCL promote more rapid hydrolytic softening in line with previous studies on hydrolytic degradation of PCL blends^33,63,64^.

### Mechanical window of electrospun PCL fibers for tissue engineering applications

**Fig. 10:**
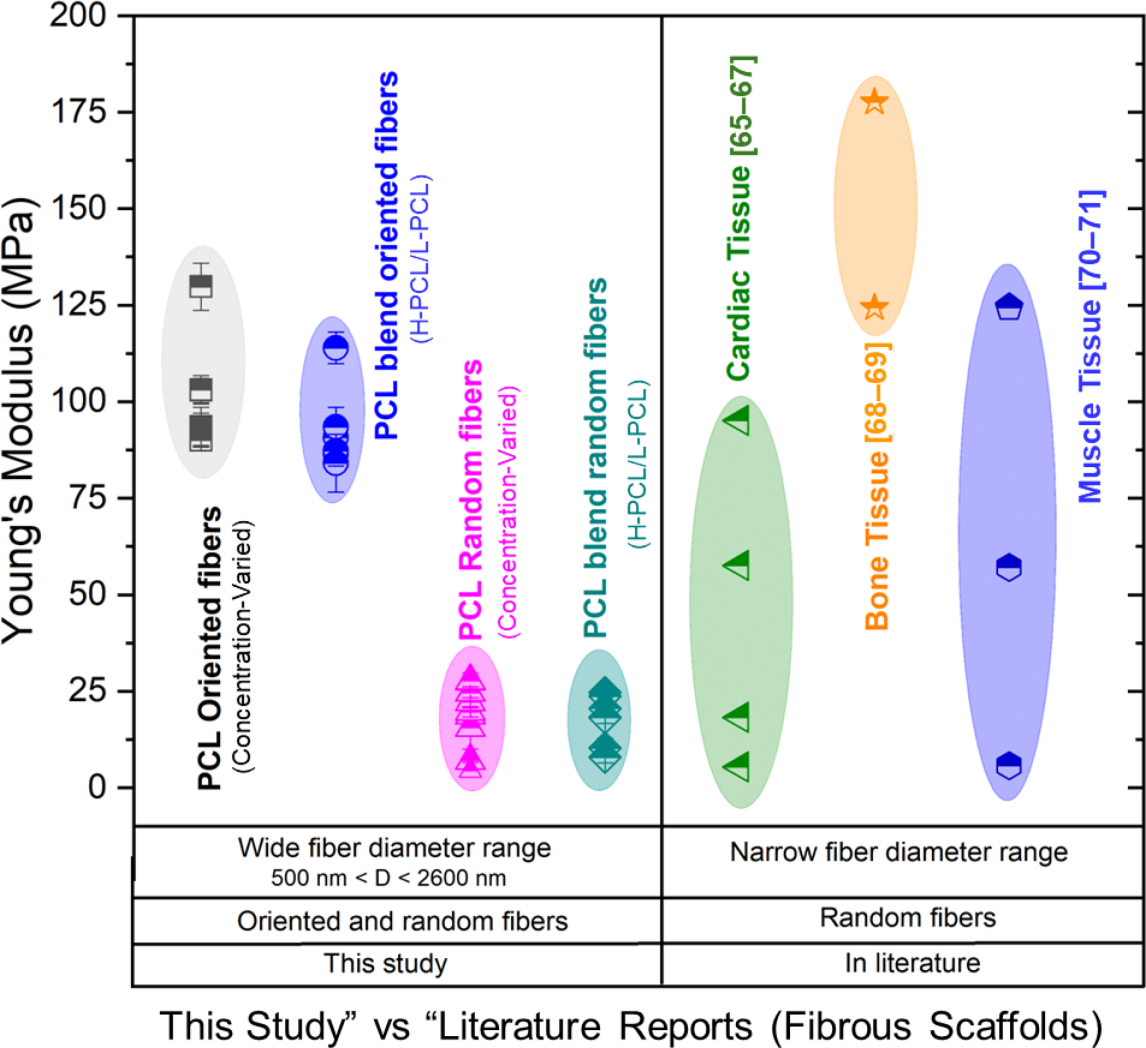
Comparative Young’s modulus of electrospun PCL fibrous scaffolds. The left panel shows the mechanical range achieved for scaffolds fabricated in this study, including different PCL systems and fiber configurations. The right panel presents representative literature-reported Young’s modulus values of electrospun PCL-based scaffolds developed for cardiac, bone, and muscle tissue engineering applications. Each literature data point corresponds to an independent published study.

Figure 10 summarizes the Young’s modulus of the electrospun PCL scaffolds developed in this study in comparison with representative literature-reported electrospun PCL-based scaffolds for cardiac, bone, and muscle tissue engineering applications. The left panel presents the mechanical range achieved in the present work, while the right panel compiles independent data points from published studies targeting specific biomedical applications.

The scaffolds fabricated in this study span a broad mechanical window (approximately ~ 5–130 MPa), encompassing both low-stiffness and relatively high-stiffness regimes within a single polymer system. When positioned against literature-reported values, the produced fibers demonstrate mechanical compatibility with ranges previously explored for several soft and semi-load-bearing tissue engineering contexts.

For cardiac applications, electrospun PCL-based scaffolds reported in the literature generally fall within the low-to-intermediate MPa range, extending in some cases to several tens of MPa depending on scaffold design. The modulus values achieved in the present study overlap with and extend across this reported window, indicating that the developed fibrous constructs fall within stiffness regimes relevant to cardiovascular patch and supportive scaffold research^65–67^.

In bone tissue engineering, electrospun PCL scaffolds are commonly employed for non-load-bearing membranes, peri-implant interfaces, and regenerative barrier applications, where reported Young’s modulus values typically range from several MPa to a few tens of MPa. The higher-modulus scaffolds produced here approach and, in some cases, exceed these values, suggesting potential suitability for mechanically reinforced fibrous membranes and interface-regeneration applications rather than bulk load-bearing bone replacement^68,69^.

For muscle tissue engineering, literature-reported electrospun PCL scaffolds generally exhibit low-to-intermediate MPa stiffness, consistent with soft tissue mechanical environments. The lower and intermediate modulus scaffolds developed in this work align well with these reported ranges, while the availability of higher stiffness variants expands the potential for mechanically tuned anisotropic constructs^70,71^.

Overall, the comparative analysis highlights that the electrospun PCL scaffolds developed in this study cover and bridge mechanical regimes reported for multiple biomedical applications. This broad tunability within a single material platform underscores the central motivation of the present work: to systematically tailor the mechanical properties of fibrous scaffolds to expand their applicability across diverse tissue engineering contexts. By situating the achieved mechanical range alongside established literature values, the figure emphasizes the translational relevance of the developed scaffolds and their potential adaptability for application-specific requirements.

## Conclusions

This study systematically investigated the structure–property relationships of electrospun poly(ε-caprolactone) (PCL) fibers by examining the combined effects of fiber orientation, polymer concentration, molecular-weight blending, and environmental exposure on mechanical performance. Among the investigated parameters, fiber orientation emerged as the dominant factor controlling mechanical behavior. Oriented fibers exhibited significantly higher Young’s modulus (~ 90–140 MPa) and tensile strength (~ 40–100 MPa), whereas randomly deposited fibers showed substantially lower stiffness (~ 6–30 MPa) but exceptional extensibility, reaching strains of up to ~ 1000%. These results highlight the inherent trade-off between stiffness and ductility in electrospun fibrous networks and demonstrate that fiber alignment is critical for achieving mechanically robust scaffolds. Polymer concentration and the resulting fiber diameter further influenced mechanical performance. Oriented fibers achieved maximum stiffness at intermediate polymer concentrations (~ 10–12% w/v) and smaller diameters (~ 550–600 nm), whereas increasing concentration to ~ 20–22% and diameters to ~ 750–850 nm resulted in reduced modulus. In contrast, random fibers exhibited larger diameters (~ 1700–2600 nm) and required higher polymer concentrations (~ 20% w/v) to reach moderate stiffness (~ 30 MPa). Stress at break followed similar trends, with oriented fibers sustaining stresses of ~ 40–100 MPa compared to ~ 5–25 MPa for random fibers. Molecular-weight blending of high-molecular-weight PCL (H-PCL) and low-molecular-weight PCL (L-PCL) provided an additional strategy to tune fiber morphology and stiffness. Oriented polyblend fibers exhibited modulus values of ~ 76–108 MPa, with peak stiffness occurring at intermediate H-PCL fractions (~ 50–60%) and smaller diameters (~ 650–700 nm). Random fibers displayed lower modulus (~ 6–27 MPa) and required higher H-PCL content (~ 80–100%) to achieve moderate stiffness. These results demonstrate that molecular-weight composition influences mechanical response differently depending on fiber alignment. Environmental exposure experiments revealed strong sensitivity of PCL fibers to acidic conditions. After 24 h soaking in acetic acid/water and formic acid/water mixtures, Young’s modulus decreased from ~ 90 MPa to ~ 60 MPa, with formic acid inducing faster degradation and dissolution at lower concentrations (~ 65–70% FA) compared with acetic acid (~ 70–75% AA). Increasing temperature further accelerated degradation, reducing modulus from ~ 80 MPa to ~ 50 MPa in 50% FA/H₂O as temperature increased toward 35–40 °C. Under physiologically relevant conditions, however, electrospun PCL fibers exhibited significantly greater stability. During soaking in PBS (pH 7.4, 37 °C) for 7 days, pure H-PCL fibers maintained relatively stable mechanical properties, whereas blends with lower H-PCL content experienced gradual softening. For example, oriented fibers with 50% H-PCL exhibited ~ 6% modulus loss after 1 day and ~ 20% after 7 days, while random fibers with 40% H-PCL showed larger reductions of ~ 15% and ~ 30%, respectively. These results indicate that both higher molecular weight and fiber alignment improve resistance to hydrolytic degradation.

Overall, the electrospun scaffolds produced in this study span a broad mechanical window of approximately ~ 5–130 MPa. When compared with literature-reported electrospun PCL scaffolds for cardiac, bone, and muscle tissue engineering, this stiffness range overlaps with and bridges multiple application-relevant regimes. These findings demonstrate that fiber orientation, polymer concentration, and molecular-weight blending can be strategically combined to engineer electrospun PCL scaffolds with tunable mechanical properties and controlled environmental stability, providing a versatile platform for designing fibrous materials tailored to diverse biomedical and tissue engineering applications.

## Data Availability

Data will be available from the authors upon request.
